# Prevalence and Associated Factors of Precancerous Cervical Lesions among Women in Ethiopia: A Systematic Review and Meta-Analysis

**DOI:** 10.4314/ejhs.v31i1.21

**Published:** 2021-01

**Authors:** Dereje Zena, Berhanu Elfu, Kebadnew Mulatu

**Affiliations:** 1 Family Guidance Association of Ethiopia, North West Branch; 2 Bahir Dar University

**Keywords:** Precancerous Cervical Lesions, Cervical Cancer, Cancer of the Uterine Cervix, Cervical Neoplasms, Cervix Neoplasms

## Abstract

**Background:**

Cervical cancer remains the most common cancer of women worldwide. Its burden is more serious in developing countries. It is also the second common cancer deaths of women in Ethiopia followed by breast cancer. The aim of this study was to determine the pooled prevalence and associated factors of precancerous cervical lesions among women in Ethiopia.

**Methods:**

We systematically searched published and unpublished articles reported from 2010 to 2019 using a comprehensive search of electronic databases including PubMed and Google scholar for grey literature from August 1 to September 1, 2019. The methodological qualities of included studies were evaluated using Joanna Briggs Institute meta-analysis of Statistics Assessment. The pooled prevalence estimate was calculated using MedCalc software-version 19.0.7, and the pooled odd ratios for predictors was calculated using RevMan software version 5.3.

**Results:**

The pooled prevalence of precancerous cervical lesions among women in Ethiopia was 13.4% (95% CI:10.63% 16.37%). Statistically significant heterogeneity between studies was detected (I^2^=83.1%, P < 0.001). Among all measured associated factors: numbers of women life time sexual partners > 1, OR=2.5 (95% CI:3.70,4.76), being HIV positive women, OR=2.4 (95% CI:1.33,4.61) and women having history of STI, OR=2.0 (95% CI:1.02,3.87) had statistically significant association with precancerous cervical lesions among women in Ethiopia

**Conclusion:**

The pooled prevalence of precancerous cervical lesions among women in Ethiopia was high as compared to the 5-year worldwide cervical cancer prevalence. Women having more than one life time sexual partners, being HIV positive women and women having history of STI had a statistically significant association with precancerous cervical lesions.

## Introduction

One of the major problems that threaten the developing countries is cervical cancer which in fact is a serious threat for the global community (1). Cervical cancer is a cancer arising from the cervix. It is due to the abnormal growth of cells and can spread to other parts of the body. Typically, no symptoms are seen at an early stage. Later symptoms may include abnormal vaginal bleeding, pelvic pain or pain during sexual intercourse (2).

Precancerous cervical lesions go through many stages and take many years to develop into cervical cancer. They become cancer when the abnormal cells spread below the epithelial layer down into the deeper tissues of the cervix (1).

Approximately 311 000 women died of cervical cancer globally in 2018 (1). It is estimated as the fourth most frequent cancer among leading causes of death for women worldwide. It represents 7.5% of all female cancer deaths, more than 85% of these deaths occurring in low and middle-income countries (1).

The 5-year prevalence of cervical cancer in sub-Saharan Africa was 27.6% with an incidence rate of 26% and 23% of mortality (3). Its prevalence across African countries ranges from 12% to 46% (4).

Cervical cancer is the second common cancer deaths of women in Ethiopia followed by breast cancer (5). According to the WHO data published in 2017, cervical cancer accounts for 0.78% of total deaths in Ethiopia. That is about 1 death due to cervical cancer of every 128 deaths or 14 people die of cervical cancer each day or an average of 1 death occurs every 2 hours(5).

Precancerous lesion is preventable through periodic screening and early detection of lesions before progress to cancer. It can be treated easily by cryotherapy using freezing gas (liquid nitrogen), Loop electrosurgical excision procedure, Laser treatment and a beam of laser light. Thus, early treatment of precancerous cervical lesion can help women avoid getting of cervical cancer (6) (7).

WHO has approved and recommended vaccines against HPV 16 and 18 for use in many countries including Ethiopia (1). The vaccine is widely administered in rich countries while countries with the highest burden of cervical cancer in Africa are covering late (10).

Ethiopia launched Human Papilloma Virus (HPV) vaccination pilot project in December 2015 in Oromia and Tigray regions. The targated groups were adolescent girls of age from 9–13 years (9).

## Methods and Materials

**Study design and setting**: In this systematic review and meta-analysis, we assessed observational studies conducted at health facility level on precancerous cervical lesions among women in Ethiopia from 2010 to 2019. Ethiopia is one of the east African countries situated in the horn of Africa having a total population of 109,616,652 and 50.2% of the female population, 56.2 years (53.6-men, 58.8-women). The country has a federal system of governance with nine regional states and two chartered cities (1, 11).

**Searching strategy**: The presence of systematic review protocol on the topic of prevalence and associated factors of precancerous cervical lesions among women in Ethiopia was checked via searching different databases include PubMed, Google scholar; Joanna Briggs Institute (JBI) databases, the national health center review and dissemination databases and Prospero for systematic review. As far as our search strategy is concerned, there was no systematic review conducted on our topic of interest. Thus, the actual search was conducted from August 1, 2019 to September 1, 2019. The electronic databases searched include PubMed, Congress Library, Medline (TR), Web of Science core collection and Google scholar. In order to minimize time-lag bias, the searching process was updated on January 12, 2020. Our search strategy focused on all published and unpublished observational studies with epidemiological data on the prevalence and associated factors of precancerous cervical lesions among women in Ethiopia. Combinations of Mesh terms such as “Prevalence OR Frequency AND Associated Factors AND Cancer of Cervix OR Cancer of the Cervix OR Cervical OR Cancer of the Uterine Cervix OR Uterine Cervical Cancer OR Cervical Intraepithelial Neoplasia OR Cervical Cancer OR Cervix Cancer OR Cervical Neoplasms OR Uterine Cervical Neoplasms OR Cervix Neoplasms OR Cervix OR Uterine Cervical Cancer OR Tumour of the Uterine Cervix OR Uterine Cervical Dysplasia AND Girls OR Woman OR Women's Groups AND Ethiopia” were used. These key terms were combined using Boolean operators “AND/OR” to narrow the search. Two authors (DZ and BE) conducted the searching process. An End Note software version X 5 was used to manage references.

**Inclusion criteria**: The inclusion criteria for this study were:

1. **Study design:** all observational studies conducted at a health facility level to assess the prevalence or associated factors of precancerous cervical lesions among women residing in Ethiopia;
2. **Publication status:** those published articles or grey literatures reported from 2010 to 2019;
3. **Language:** articles reported in the English language;
4. **Study quality:** those primary studies which scored ≥ 60% of the Joanna Briggs Institute (JBI) criteria for assessing the quality of primary studies included in the meta-analysis; and
5. **The outcome of interest**: precancerous cervical lesion positive result diagnosed by any methods.

**Data extraction:** The retrieved data were screened independently by two reviewer authors (DZ and BE) to verify studies that possibly met the inclusion criteria. Any disagreement was resolved through discussion with a third reviewer (KM). The third reviewer mediated any issues that remained unresolved. Data were extracted based on standardizing data extraction tools adapted from the JBI Meta-analysis of Statistics Assessment and Review Instruments (MASARI) (12).

Appropriate critical appraisal checklist of Meta-analysis of Observational Studies in Epidemiology (MOOSE) for systematic reviews (13) and the Preferred Reporting Items for Systematic Reviews and Meta-analyses (PRISMA) statement for reporting systematic reviews and meta-analyses were used to assess the overall methodological quality of included studies (14). End Note X 5 citation manager was used to import and handle all searched articles and remove duplicates. Each study data was extracted following study characteristics (year of publication, women demographics and baseline characteristics, study setting, number of cases enrolled, study population and study designs), prevalence and associated factors of precancerous cervical lesions. The data were extracted from the included studies independently by the two authors and the completeness of the retrieved data was checked by the two authors together (Table 1, Table 2).

**Table 1:** Summary of eligible articles included to review the pooled prevalence and associated factors of precancerous cervical lesion among women in in Ethiopia

ID	Author name and publication	Tittle of the study	Study design	Overall quality
10	Mesele B. etal, 2015	Risk Factors Associated with Invasive Cervical Carcinoma among Women Attending Jimma University Specialized Hospital, Southwest Ethiopia	Case control	8/10
16	Deksissa etal, 2015	Prevalence and factors associated with VIA positive result among clients screened at Family Guidance Association of Ethiopia, south west area office, Jimma model clinic, Jimma, Ethiopia	Cross-sectional	7/9
35	Getinet M. etal, 2015	Prevalence and predictors of Pap smear cervical epithelial cell abnormality among HIV-positive and negative women attending gynecological examination in cervical cancer screening center at Debre Markos referral hospital, East Gojjam, Northwest Ethiopia	Comparative Cross-sectional	6/8
47	Kassa R. 2018	Risk factors associated with precancerous cervical lesion among women screened at Marie Stops Ethiopia, Adama town, Ethiopia	Case control	8/10
50	Leyh-B. etal, 2014	Cervical human papillomavirus prevalence and genotype distribution among hybrid capture 2 positive women 15 to 64 years of age in the Gurage zone, rural Ethiopia	Cross-sectional	7/8
61	Gebreheat G. etal, 2018	Factors associated with cervical precancerous lesions among women screened for cervical cancer in Addis Ababa, Ethiopia	Case control	8/10
69	Hailemariam T. etal, 2017	Prevalence of Cervical Cancer and Associated Risk Factors among Women Attending Cervical Cancer Screening and Diagnosis Center at Yirgalem General Hospital, Southern Ethiopia	Cross-sectional	7/9
76	Misgina etal, 2016	Prevalence of precancerous cervical lesion and associated factors among women in North Ethiopia	Cross-sectional	7/9
85	Ali etal, 2019	Burden and genotype distribution of high-risk Human Papillomavirus infection and cervical cytology abnormalities at selected obstetrics and gynecology clinics of Addis Ababa, Ethiopia	Cross-sectional	7/9

**Table 2:** Summary of eligible articles included to review the pooled prevalence and associated factors of precancerous cervical lesion among women in in Ethiopia

ID	Author name and publication	Total sample size	Prevalence	Statistically significant associated factors
10	Mesele B. etal, 2015	180 (60:120)	NA	Women: 40–59 years, AOR= 4.7 & 95%CI (2.3, 9.6) Had >1 husband, AOR= 2.0; 95%CI (1.0, 3.9) and Had > 4 children, AOR =10.3, 95% CI (3.6, 29.0)
16	Deksissa etal, 2015	334	43(12.9%)	• Sexual intercourse < 16 years, OR=2.2,95% CI (1.1, 4.3)
35	Getinet M. etal, 2015	391	55(14.1%)	HIV+ women, AOR =1.9, 95 % CI (1.1, 3.4) Multiple sexual partnership, AOR =3.2, 95 % CI (1.1, 10.0) First sexual contact <15 years, AOR =5.2, 95 % CI (1.5, 17.9) and Long term oral contraceptive pills use, AOR =11.9, 95% CI (2.1, 16.7)
47	Kassa R. 2018	164 (55:109)	NA	Use of oral contraception OR=2.342, 95CI (1.1, 4.9) History of STI, A OR= 2.485, 95CI (1.19 5.2) and Age at1st sexual intercourse <15 years, AOR=6.70, 95CI % (1.73, 10.12)
50	Leyh-B. etal, 2014	537	86(16.1%)	Widowed AOR =1.85, 95%CI (1.0, 3.4) and Had >1 lifetime sexual partners AOR =1.83, 95%CI (1.0, 3.2)
61	Gebreheat G. etal, 2018	343 (114:229)	NA	40–49 years, AOR=2.55, 95% CI (1.5, 4.2) having history STI AOR=3.20, 95% CI (1.2, 8.1) and had >/=2 lifetime sexual partners, AOR=2.17, 95% CI (1.0, 4.7)
69	Hailemariam T. etal, 2017	1945	321(16.5%)	With HIV, AOR=9.03, 95%CI (4.5, 18.0), had history of STI, AOR=8.36:95% CI (5.6, 12.4) and age at first sexual intercourse, AOR=8.97,95%CI (5.6, 14.4)
76	Misgina etal, 2016	342	23(6.7%)	
85	Ali etal, 2019	366	50(13.7%)	Unemployed AOR=9.17, 95%CI (1.6, 52.2) Positive AOR=5.73, 95%CI (1.1,30.9) and Resided out of Addis Ababa AOR=8.12, 95%CI (1.1, 57.9)

**Risk of bias (quality) assessment**: Two review authors (DZ & BE) assessed the methodological quality primarily studies independently using appropriate critical appraisal checklist of Meta-analysis of Observational Studies in Epidemiology (MOOSE) before eligible articles were included. Moreover, the completeness of outcome data and other sources of bias were effectively assessed by two review authors (DZ & BE) using Risk Of Bias In Nonrandomized Studies of Interventions (ROBINS-I) with detailed guidance to make domain-level judgments about risk of bias (15). Any disagreements between two review authors (DZ & BE) were resolved by discussion and involvement of a third review author as the mediator (KM). To minimize time-lag bias, the search was updated on August 30, 2019. Those primary studies (which had a score of one point each) which scored ≥60% of the Joanna Briggs Institute (JBI) criteria were included in the metaanalysis. During our study, methodological quality assessment and scoring, one disagreement (1/9) happened between the primary (DZ) and secondary (BE) review authors. However, the difference was resolved by the third review author (KM) through discussion. Moreover, a funnel plot was used to assess publication bias.

## Results

**Figure 1:**
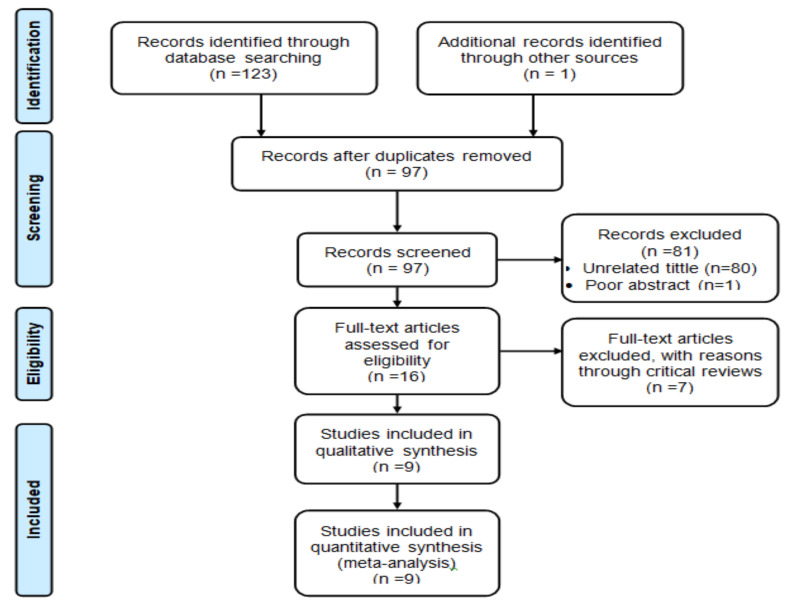
Flow diagram of included and excluded articles to review the prevalence and associated factors of precancerous cervical lesion among women in Ethiopia.

**Study Description**: A total of 124 articles was retrieved and screened. After removing 27 duplicates, 97 articles were screened by title and abstract; 80 articles were excluded by unrelated tittle and one article was excluded by poor abstract contents. Then, 16 full-text articles were assessed for eligibility, but seven full-text articles were excluded with reason through a critical review process. Finally, a total of nine full-text articles (five cross-sectional, one comparative cross-sectional and three case-control studies) met the inclusion criteria for this study (Figure 1).

**The pooled prevalence estimates of precancerous cervical lesions among women in Ethiopia**: Six full-text articles conducted at health facility level with a total sample size of 3915 were included to compute the pooled prevalence estimate of precancerous cervical lesions among women in Ethiopia. Therefore, the highest prevalence was observed in a study done in southern Ethiopia (16.5 %) (20) whereas the lowest point estimate was observed at a study conducted in northern Ethiopia in 2017 (6.7%) (21). Statistically significant heterogeneity between studies was also detected (I^2^=83.1%, p<0.001). Due to the fat that the combined studies were less than ten, this heterogeneity cannot be explained by running a meta-regression.

**Figure 2:**
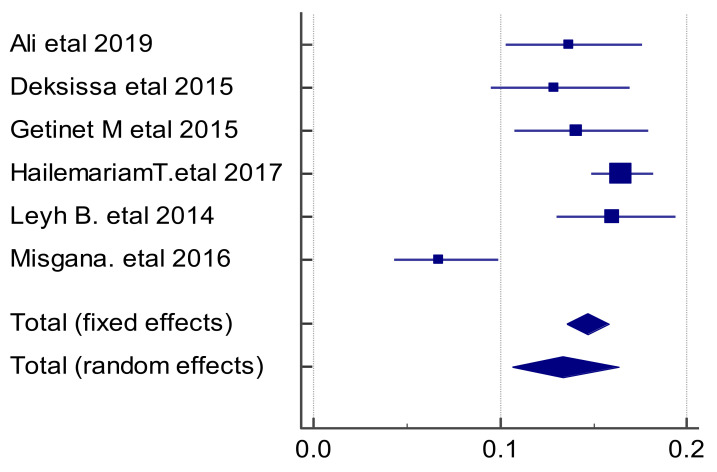
The pooled prevalence of precancerous cervical lesion among women in Ethiopia.

This study based meta-analysis forest plot with random effects model discovered that more weight was given for a study conducted in Yirgalem General Hospital in 2017 (20). Therefore, the combined point estimate of precancerous cervical lesions was found to be 13.4% (95% CI:10.63%,16.37%) (Figure 2).

Publication bias was indicated by funnel plot asymmetry. Thus, visual inspection of the funnel plot confirmed that the plot was asymmetrical.

**Factors associated with precancerous cervical lesions among women in Ethiopia**: All nine eligible full-text articles were used to access associated factors of precancerous cervical lesions among women in Ethiopia. Of these three studies were conducted in the Oromia region followed by two studies conducted in Southern Nations Nationalities and Peoples Region and Addis Ababa and one study each was conducted in Amhara and Tigray regions.

Three studies were eligible to conduct women's marital status subgroup meta-analysis (20–22). The study done at Yirgalem General Hospital in 2017 had more effect size and weight to predict the overall effect estimate (20). Its 95% confidence interval did not overlap the line of no effect (null value). The 95% confidence intervals of all other studies overlap 1 or the null value (21, 22). However, the 95% CI of the overall effect estimate did overlap the line of no effect (null value). Therefore, the overall effect estimates of women's marital status were OR=0.3 (95 % CI:0.14,0.75).

From studies eligible to proceed with women's age subgroup meta-analysis (22–27), a study conducted in Addis Ababa in 2019 (22) had more effect size to pull the overall effect estimate to the left (favors to the bad event). The 95% CI of this study did not overlap the line of no effect (null value) like studies conducted in Addis Ababa (24) and Jimma (27) in which both had the lowest effect to predict the overall effect estimate respectively. More weight (19.2%) was given for studies conducted in Addis Ababa (24) and the Gurage zone (23). Therefore, the overall effect estimates of women's age were OR=1.4 (95 % CI:0.65,3.12).

Except for one study (20), the 95%CI of all other studies overlap the line of no effect at a study level (22, 24, 25). As a result, the overall effect estimate touches the line of no effect. Therefore, the overall effect estimates of women's educational background were OR=0.04 (95 % CI: 0.09,1.72).

Among three eligible studies (20, 22, 24), the study conducted at Yirgalem General Hospital in 2017 had more effect size and highest weight to forecast the overall effect estimate (20). Yet, a study conducted in Addis Ababa in 2018 had the lowest effect size and weight (22). Due to its higher weight (35.8%) and more effect size, the study done at Yirgalem General Hospital pulls the overall effect estimate towards the better event (favors to negative) (20). The 95%CI of the overall effect estimate did overlap the line of no effect (null value). Therefore, the overall effect estimates of women's occupation were OR=1.1 (95 % CI: 0.24,4.89).

In this analysis (20, 27), more effect size was observed at a study conducted in Jimma (27) whereas more weight (52.7%) was given for a study conducted at Yirgalem General Hospital (20). However, the 95% CI of overall effect estimate of women's residence, OR=1.4 (95% CI:0.37,5.58) overlaps the line of no effect (null value).

Income was another important variable to predict precancerous cervical lesion distribution among women in Ethiopia. The 95% confidence intervals of all the studies did not overlap the line of no effect (null value) (24, 27), and the 95% confidence intervals of the overall effect estimate did not overlap the line of no effect (null value). Therefore, the overall effect estimates of women's income were OR=1.8 (95 % CI:1.19,2.65)

As the forest plot of all studies (20, 25, 26) indicates that the overall effect estimates of contraceptive utilization did not touch the no effect line, or null value, except one study (20), both crossed the line of no effect (null value). Therefore, the overall effect estimates of contraceptive utilization were OR=2.6 (95 % CI: 2.13,3.24).

**Figure 3:**
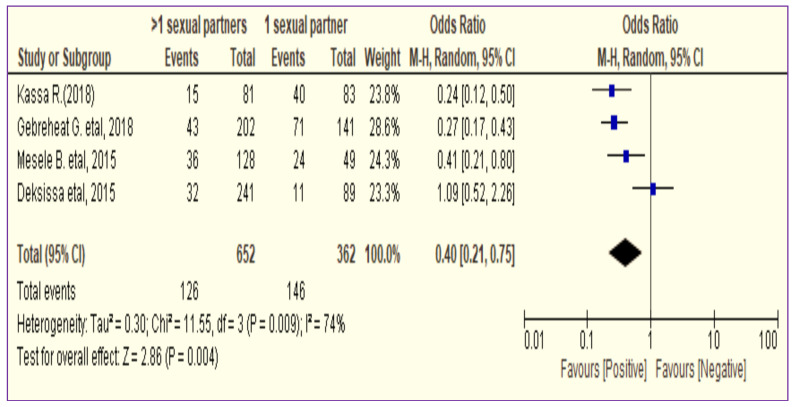
The association between residence and precancerous cervical lesion among women in Ethiopia (n=4).

In all studies (24, 25, 27), except one study done in Jimma (26), the 95% confidence intervals did not touch the line of no effect (null value) (24, 25, 27). As a result, their combined effect pulls the overall effect estimate towards the left (bad event/cervical cancer episode). Thus, the overall pooled prevalence estimates of precancerous cervical lesions prevalence between women having > 1 life time sexual partners and women who had 1 lifetime sexual partner was OR=0.4 (95% CI:0.21,0.75) (Figure 3).

**Figure 4:**
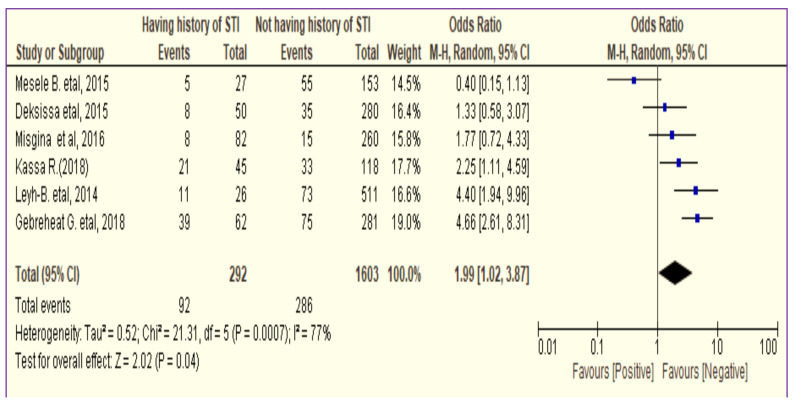
The association between history of STI and precancerous cervical lesion among women in Ethiopia (n=6).

Like other variables, having previous history of STI can predict the prevalence of precancerous cervical lesion among women in Ethiopia (21, 23–27). Except three studies (21, 26, 27), the 95% CI of all other studies did not overlap the null value (23–25). Therefore, the overall effect estimates of cervical cancer between history of STI and precancerous cervical lesion among women were OR=2.0 (95% CI:1.02,3.87) (Figure 4).

The forest plot of women's HIV Status (20, 21, 24–26, 28) shows that the highest effect size but the least weight (7.4%) was given for a study done in North Ethiopia (21). The 95% confidence intervals of studies conducted at Debre Markos Referral Hospital, Addis Ababa and Yirgalem General Hospital did not overlap the line of no effect (null value) (20, 24, 28) like the pooled prevalence estimate of precancerous cervical lesions between HIV plosive and negative women. Therefore, the overall effect estimates of cervical cancer between HIV status and precancerous cervical lesion among women were OR=2.2 (95% CI:1.10,4.33).

In all studies (22, 24, 26, 27), except studies done in the Gurage zone (23) and Adama town (25), the 95% CIs crossed the no effect line (null value). As result, the overall effect estimate (the diamond) of age at first sexual intercourse also overlaps the no effect line. Therefore, the overall effect estimates of cervical cancer between age at first sexual intercourse and precancerous cervical lesion among women were OR=1.0 (95% CI:0.47,2.02).

The 95% confidence intervals of both studies (24, 27) overlap the line of no effect (null value). Therefore, the overall effect estimates of cervical cancer between ever had cervical cancer screening and precancerous cervical lesion among women were OR=0.7 (95% CI: 0.45,1.24).

## Discussion

The pooled prevalence estimate of precancerous cervical lesions among women in Ethiopia was (13.4%). The highest prevalence was observed in a study done in southern Ethiopia (16.5%) (20) whereas the lowest point estimate was observed at a study conducted in northern Ethiopia in 2017 (6.7%) (21). This pooled prevalence estimate of precancerous cervical lesions was comparable with studies conducted in Egypt (10.4%) (29), China (15.5%) (30) and Latin America (16.1%) (31). However, it was lower than the study conducted in Sudan (24.0%) (32) and the 5-year worldwide cervical cancer prevalence (9.0%) in 2012 (3) but higher than the study done in Qatar (8.1%) (33). These differences might be due to socio-economic variations.

There was a variation of evidence reported on the effect of associated factors on precancerous cervical lesions like women's age, education, occupation, residence and age at first sexual intercourse. Some studies revealed that they had a statistically significant association with cervical cancer while others did not show a statistically significant association with cervical cancer at their study levels. This might be due to a difference in sample size, variance, methodology, study populations and reliability of the outcome measures at each study level.

The odd of precancerous cervical lesions was 68.0 % lower among married, widowed and divorced women than single women. It might be that all single women were not protected from the risk of cervical cancer infection.

This study did not show the overall effect difference between prevalence of precancerous cervical lesions and women's education like first years of sexual intercourse debut and cervical cancer screening practices.

Being unemployed women did not have a clear difference effect on precancerous cervical lesions as compared to employed women. Fewer episodes of precancerous cervical lesions were observed among women who did not have an experience of using modern contraceptives than users (Figure11). However, more episode of cervical cancer was observed among women who ever had >1 lifetime sexual partners than women who ever had 1 lifetime sexual partner (Figure 3).

The better outcome of precancerous cervical lesions was observed among women who did not have history of STI than women having history of STI. (figure 4). Moreover, precancerous cervical lesion occurred less frequently among HIV negative women than HIV positive women (OR < 1).

Statistically significant heterogeneity between studies was also detected (I^2^=83.1%, P < 0.001). This might show that studies were inconsistent due to a reason other than chance.

This study was not conducted without limitations. Some of its limitations are failure to show subgroup analysis due to a small number of studies included and failure to show some important findings due presence of few important missing data at study levels.

In conclusion, the pooled prevalence of cervical cancer among women in Ethiopia was higher than the 5-year worldwide cervical cancer prevalence (3). Some variables like income, being HIV positive, previous STI history, more than one number of lifetime sexual partners and prolonged uses of modern contraceptives had a statistically significant association with the pooled prevalence estimates of cervical cancer among women in Ethiopia. There was also a variation of cervical cancer reports across studies in the country.

Therefore, unbiased reports on prevalence and associated factors of precancerous cervical lesions is important for other researchers to enhance future studies. If so policymakers and practitioners can easily understand the burden of cervical cancer in Ethiopia for better prevention, diagnosis, and treatment of the disease.

## References

[1] WHO (2019). Human papillomavirus (HPV) and cervical cancer fact sheet.

[2] Athinarayanan S, Srinath M, Kavitha R (2016). Detection and Classification of Cervical Cancer in Pap Smear Images using EETCM, EEETCM & CFE methods based Texture features and Various Classification Techniques. IJSRSET.

[3] Ahmad A Cervical cancer statistics and Facts.

[4] Smith JS MA, Rana RK, Pimenta JM (2008). Age-specific prevalence of infection with human papillomavirus in females: a global review. Journal of Adolescent Health.

[5] WHO (2017). Cervical Cancer in Ethiopia.

[6] Wright TC, Massad LS, Dunton CJ, Spitzer M, Wilkinson EJ, Solomon D (2007). 2006 consensus guidelines for the management of women with abnormal cervical cancer screening tests. American journal of obstetrics and gynecology.

[7] Mishra GA, Pimple SA, Shastri SS (2011). An overview of prevention and early detection of cervical cancers. Indian journal of medical and paediatric oncology: official journal of Indian Society of Medical & Paediatric Oncology.

[8] Wright TC, Kuhn L (2012). Alternative approaches to cervical cancer screening for developing countries. Best practice & research Clinical obstetrics & gynaecology.

[9] WHO Ethiopia Launchs a Pilot Human Papilloma Virus Vaccination Project.

[10] Ferlay J, Ervik M, Lam F, Colombet M, Mery L, Piñeros M (2018). Global cancer observatory: cancer today.

[11] Degefa T (2019). Ethiopia: When does population become an economic asset?. Pula: Botswana Journal of African Studies.

[12] Munn Z MS, Lisy K, Riitano D, Tufanaru C (2015). Methodological guidance for systematic reviews of observational epidemiological studies reporting prevalence and incidence data. Int J Evid Based Healthc.

[13] Stroup DF B, Morton SC, Olkin I, Williamson GD, Rennie D (2000). Meta-analysis of observational studies in epidemiology. JAMA: the journal of the American Medical Association.

[14] Liberati AA, Tetzlaff J, Mulrow C, Gotzsche PC, Ioannidis JP (2009). The PRISMA statement for reporting systematic reviews and metaanalyses of studies that evaluate healthcare interventions:explanation and elaboration. PLoSMed.

[15] Schünemann HJ TP, Reeves BC (2013). Nonrandomized studies as a source of complementary, sequential or replacement evidence for randomized controlled trials in systematic reviews on the effects of interventions. Research Synthesis Methods.

[16] Barendregt JJ DS, Lee YY, Norman RE, Vos T (2013). Meta-analysis of prevalence. J Epidemiol Commun Health.

[17] (2011). (RevMan): Version 5.1.

[18] GW C (1954). The combination of estimates from different experiments. Biometrics.

[19] Egger M DSG, Schneider M, Minder C (1997). Bias in meta-analysis detected by a simple, graphical test. BMJ.

[20] Hailemariam T, Yohannes B, Aschenaki H, Mamaye E, Orkaido G, Seta M (2017). Prevalence of cervical Cancer and associated risk factors among women attending cervical Cancer screening and diagnosis Center at Yirgalem General Hospital, southern Ethiopia. J Cancer Sci Ther.

[21] Misgina KH, Belay HS, Abraha TH (2017). Prevalence of precancerous cervical lesion and associated factors among women in North Ethiopia. Journal of Public Health and Epidemiology.

[22] Ali KE, Mohammed IA, Difabachew MN, Demeke DS, Haile T, ten Hove R-J (2019). Burden and genotype distribution of highrisk Human Papillomavirus infection and cervical cytology abnormalities at selected obstetrics and gynecology clinics of Addis Ababa, Ethiopia. BMC cancer.

[23] Leyh-Bannurah S-R, Prugger C, de Koning MN, Goette H, Lellé RJ (2014). Cervical human papillomavirus prevalence and genotype distribution among hybrid capture 2 positive women 15 to 64 years of age in the Gurage zone, rural Ethiopia. Infectious agents and cancer.

[24] Teame H, Addissie A, Ayele W, Hirpa S, Gebremariam A, Gebreheat G (2018). Factors associated with cervical precancerous lesions among women screened for cervical cancer in Addis Ababa, Ethiopia: A case control study. PloS one.

[25] Kassa RT (2018). Risk factors associated with precancerous cervical lesion among women screened at Marie Stops Ethiopia, Adama town, Ethiopia 2017: a case control study. BMC research notes.

[26] Deksissa ZM, Tesfamichael FA, Ferede HA (2015). Prevalence and factors associated with VIA positive result among clients screened at Family Guidance Association of Ethiopia, south west area office, Jimma model clinic, Jimma, Ethiopia 2013: a cross-sectional study. BMC research notes.

[27] Bezabih M, Tessema F, Sengi H, Deribew A (2015). Risk factors associated with invasive cervical carcinoma among women attending Jimma University Specialized Hospital, Southwest Ethiopia: A case control study. Ethiopian journal of health sciences.

[28] Getinet M, Gelaw B, Sisay A, Mahmoud E A, Assefa A (2015). Prevalence and predictors of Pap smear cervical epithelial cell abnormality among HIV-positive and negative women attending gynecological examination in cervical cancer screening center at Debre Markos referral hospital, East Gojjam, Northwest Ethiopia. BMC Clinical Pathology.

[29] Shaltout MF (2014). Prevalence and type distribution of human papillomavirus among women older than 18 years in Egypt: a multicenter. International Journal of Infectious Diseases.

[30] Zhu B, Liu Y, Zuo T, Cui X, Li M, Zhang J, Yu H, Piao H (2019). The prevalence, trends, and geographical distribution of human papillomavirus infection in China: The pooled analysis of 17 million women. Cancer medicine.

[31] Bruni L, Diaz M, Castellsagué M, Ferrer E, Bosch FX, de Sanjosé S (2010). Cervical Human Papillomavirus Prevalence in 5 Continents: Meta-Analysis of 1 Million Women with Normal Cytological Findings. The Journal of Infectious Diseases.

[32] Shammat IM MZ, Alnayal MD, Elsadig M (2015). Direct visual inspection of the cervix with acetic acid for the detection of premalignant lesions. Sudan Medical Laboratory Journal.

[33] Elmi AA, Bansal D, Acharya A, Skariah S, Dargham SR, Abu-Raddad LJ, Mohamed-Nady N, Amuna P, Al-Thani AA, Sultan AA (2017). Human papillomavirus (HPV) infection: molecular epidemiology, genotyping, seroprevalence and associated risk factors among Arab women in Qatar. PloS one.

